# Relationships Among Comorbidities, Disease Severity, and Hospitalization Duration in the United States Using the Healthcare Cost and Utilization Project (HCUP) Database

**DOI:** 10.3390/jcm14030680

**Published:** 2025-01-21

**Authors:** Junse Lee, Jungmin Park

**Affiliations:** 1 School of AI Convergence, Sungshin Women’s University, Seoul 02844, Republic of Korea; junselee@sungshin.ac.kr; 2 College of Nursing, Hanyang University, Seoul 04769, Republic of Korea; 3 School of Nursing, The University of Texas, Austin, TX 78712, USA

**Keywords:** chronic condition, demographic, disease severity, discharge, hospital length of stay

## Abstract

**Background/Objectives**: Hospital length of stay (LOS) is widely analyzed and serves as a benchmark for assessing changes during hospitalization. This study introduced a method to estimate patients’ LOS and highlighted the variations in LOS among individuals with or without multiple chronic conditions (MCCs) and across different levels of disease severity, using data from the 2016 National Inpatient Sample in the United States. **Methods**: To analyze the factors influencing LOS, a multinomial logistic regression model was employed, demonstrating its effectiveness in estimating and predicting expected LOS. Factors such as demographic characteristics, MCCs, and disease severity were strongly linked to LOS. **Results**: The overall prevalence of MCCs exceeded 66%, rising to over 90% among elderly patients and more than 88% among those with severe diseases. LOS distribution was primarily concentrated within the first month following admission: over 13% of patients were discharged within a day, over 85% within a week, and more than 99% within a month. Multinomial logistic regression analysis showed that LOS was significantly influenced by age, disease severity, and the presence of MCCs. Older patients, especially those with MCCs, had significantly longer LOSs compared to younger patients without MCCs. **Conclusions**: LOS tended to increase with age and higher disease severity, particularly in patients with MCCs. Multinomial logistic regression revealed that patients over 65 and those with high disease severity (severity score 4) had significantly longer LOS. Shorter LOS was more frequent among patients under 65 years old, those without MCC, and those with low disease severity, whereas longer LOS was commonly observed in patients with MCCs or high disease severity.

## 1. Introduction

Multiple chronic conditions (MCCs), also referred to as multimorbidity, describe patients who simultaneously have three or more chronic conditions [1]. This definition is widely accepted in clinical practice and health policy documents [2,3,4]. The threshold of three chronic conditions is regarded as a valid criterion for defining multimorbidity, particularly in elderly populations [1,5,6]. According to previous studies, patients with MCCs typically incur higher healthcare costs due to the complexity of managing multiple diseases, which requires intricate treatment strategies [7,8,9]. The prevalence of MCCs varies across regions and populations, ranging from 16% to 58% in UK studies, 26% in US studies, and 9.4% among urban South Asians [10]. Additionally, as life expectancy increases, the prevalence of MCCs also tends to rise with age [11,12,13]. The number of chronic conditions a person has is closely linked to mortality rates and hospital discharge outcomes [14].

To quantify comorbidities, several standardized indexes have been developed and widely adopted in healthcare research. The Charlson Comorbidity Index (CCI) and the Elixhauser Comorbidity Index are among the most recognized tools, both predicting mortality and healthcare outcomes based on the presence of various chronic conditions. The Comorbidity–Polypharmacy Score (CPS) extends this approach by incorporating medication burden into the assessment, while the Revised Cardiac Risk Index (RCRI) is tailored to evaluating surgical outcomes. Additionally, the Case Mix Index (CMI) offers a hospital-level measure of patient complexity by accounting for the resource intensity required for different diagnoses [15,16,17,18,19]. These indexes provide valuable frameworks for analyzing the relationship between comorbidities and hospital length of stay (LOS), but they do not capture the combined effects of MCCs, demographic factors, and disease severity.

Measures of disease severity, such as the risk of mortality (ROM) and severity of illness (SOI), are part of an inpatient classification system that assigns diagnostic-related groups. These measures assess the impact of disease on resource utilization, comorbidities, and mortality [14]. The early identification of disease severity is crucial for developing optimal treatment plans, especially for patients with MCCs [20,21].

Hospital length of stay (LOS), defined as the time interval between a patient’s admission and discharge, is one of the most commonly studied outcome metrics and serves as a critical benchmark for assessing changes in patient care processes [14,22]. Identifying factors that predict LOS is a vital aspect of outcome research and quality-of-care analysis as it enables physicians and patients to anticipate both survival and LOS. Moreover, LOS significantly impacts hospital operations, influencing resource allocation, staffing, bed availability, and financial planning. A shorter LOS is often associated with reduced costs per discharge [23]. Consequently, estimating a patient’s expected LOS during treatment is highly valuable for optimizing care delivery and organizational performance.

Various methods, such as linear regression, log-normal analysis, and Markov chain models, have been used in previous studies to analyze LOS [24,25,26]. However, these studies have been criticized for not accounting for the positive skewness often observed in LOS data [27]. MCCs are frequently considered a primary factor in LOS analyses due to their prevalence and significant impact on healthcare outcomes [28]. MCCs contribute to prolonged LOS, increased in-hospital mortality, and higher healthcare burdens, especially in older patients [29]. Disease severity is also a critical predictor of in-hospital death and LOS due to its strong influence on mortality [20]. Despite this, many studies have either failed to analyze the combined effects of MCCs, demographic factors, and disease severity on LOS or have focused only on specific disease groups [21,22,23,30].

This study aims to address these gaps by employing a quantitative approach to estimate and predict LOS, examining how age, sex, the presence of MCC, and varying levels of disease severity interact to influence hospital outcomes.

## 2. Materials and Methods

### 2.1. Data and Variable Definitions

This study utilized the National Inpatient Sample (NIS) 2016 data obtained from the Healthcare Cost and Utilization Project (HCUP) database in the United States [31]. The dataset includes medical records from 1 January 2015 to 30 September 2015, covering 46 states and the District of Columbia. It represents over 97% of the U.S. population and accounts for nearly 96% of discharges from community hospitals nationwide. To minimize the bias caused by outliers, only patients without external causes of morbidity were included.

The definitions of the variables used in this study can be found in Appendix A.

### 2.2. Methods

Several descriptive statistics were employed to analyze patient characteristics, the prevalence of MCCs, and differences in LOS between patients with and without MCCs, as well as among patients with varying levels of disease severity.

To assess the factors influencing LOS, a multinomial logistic regression model, as described in [26], was utilized. In this model, LOS served as the response variable, while the predictors included AGE, SEX, MCC, ROM, and SOI.

Let $p_{i}=P\left(LOS=i\right)\;and\;\;i=1,2,3,4$. $p_{i}$ indicates the corresponding probabilities for a patient who has LOS in category LOS=i for i=1,2,3,4, respectively. In the multinomial logistic regression model, the category LOS=1 is used to designate the reference, and the other LOS categories are separately regressed against the reference. The model is given by the following equation:

$$\begin{array}{l} logit_{i}^{\left(\beta \right)}=\log\left(\frac{p_{i}}{p_{1}}\right)=\beta_{i}^{\left(0\right)}+\beta_{i}^{\left(age\right)}\left(AGE=1\right)+\beta_{i}^{\left(sex\right)}\left(SEX=1\right)+\beta_{i}^{\left(mcc\right)}\left(MCC=1\right)\\ \;\;\;\;+\sum_{j=1}^{4}\beta_{ij}^{\left(rom\right)}(ROM=j)+\sum_{j=1}^{4}\beta_{ij}^{\left(soi\right)}(SOI=j)\;\;\;\;\;,\;i=2,3,4 \end{array}$$

The regression coefficients are estimated simultaneously using the maximum likelihood method. The Wald test [32] is applied to assess statistically significant differences in the estimates, while the goodness-of-fit test from [33] is used to evaluate the model’s suitability. Each coefficient represents the change in the log-odds ratio of the response variable’s difference in a specific category compared to the reference category, corresponding to a one-level change in the respective predictor.

The following formulas are used to compute the predicted probabilities:

$$p_{i}=\frac{1}{1+\sum_{i=2}^{4}\exp\left(logit_{i}^{\left(\beta \right)}\right)}\exp\left(logit_{i}^{\left(\beta \right)}\right),\;\;\;i=2,3,4.$$

With the results obtained from model fitting, we report the estimates of the regression coefficients and show a 95% confidence interval (CI) for each estimate. Odds ratios (ORs) are reported at a 5% level of significance. For forecasting purposes, we reported an average of the probabilities for each patient characteristic corresponding to the LOS and compared patients with and without MCCs.

Further, in order to assess the assumption of multicollinearity, we calculated the Variance Inflation Factor (VIF) for each predictor variable included in the logistic regression models. The VIF values were found to be as follows: AGE (1.2), SEX (1.1), MCCs (1.3), and ROM (1.5). These values are all well below the commonly accepted threshold of 5, indicating that multicollinearity is not a significant issue in our analysis.

Additionally, the analysis in this study was conducted using R software version 4.2.0.

## 3. Results

### 3.1. Characteristics of Patients and the Prevalence of MCCs

We examined data from 748,572 patients, with over 58% being female. Most patients were under 65 years old (57%), and among them, females accounted for more than 61%. For patients aged 65 years and older, the proportion of females was lower. The proportion of females decreased with increasing disease severity. Females made up 65% of patients with level 1 severity but only 48% at level 4.

The overall rate of multiple chronic conditions (MCCs) was 66.73%, exceeding 90% in patients aged 65 or older and those with extreme disease severity. Patients with a safe severity level (coded as 0) were the smallest group (0.03%), with an MCC rate of 31.79%. Most patients had severity levels coded 1 to 3, while those with extreme severity (coded as 4) represented over 6% and had an MCC rate exceeding 88%.

**Figure 1:**
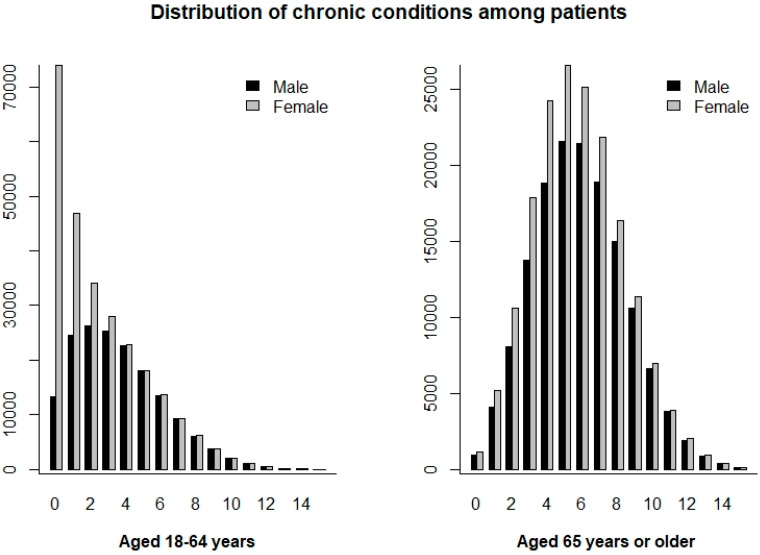
Prevalence of multiple chronic conditions (MCCs) among patients. The horizontal axis represents the number of chronic conditions, and the vertical axis represents the corresponding count.

Patient characteristics are detailed in Table 1, and the prevalence of MCCs is shown in Figure 1.

### 3.2. Differences in LOS Among Patient Characteristics

Table 2 illustrates the LOS distribution among patient characteristics, and the results from the multinomial logistic regression model are presented in Table 3. At a 5% significance level, all predictors are statistically significant. The extremely low *p*-value (approximately 2.2 × 10^−16^) from the goodness-of-fit test indicates that the model is appropriate and well-fitting. This suggests that factors such as AGE, SEX, MCC, and disease severity measures (ROM and SOI) significantly influence the length of stay (LOS) for patients.

The odds ratios (ORs) in Table 4 reveal strong associations between AGE, SEX, MCC, disease severity measures (ROM and SOI), and patients’ LOS. For instance, the likelihood of having an LOS of 2–7 days (LOS = 2) compared to 1 day (LOS = 1) decreases by 2.2% when moving from patients under 65 years (AGE = 0) to those 65 years and older (AGE = 1). The odds of having an LOS of 8–30 days (LOS = 3) or more than 30 days (LOS = 4) compared to 1 day also decrease significantly by 14.4% and 49.4%, respectively, for patients aged ≥ 65 years.

Females have higher odds of an LOS of 2–7 days or 8–30 days compared to 1 day but lower odds for an LOS of more than 1 month. For MCCs, patients with multiple chronic conditions have lower odds of having an LOS of 2–7 days, 8–30 days, or more than 30 days (by 24.1%, 9.5%, and 34.8%, respectively) compared to those without MCCs.

The risk of a longer LOS increases as severity levels rise. Specifically, the odds of having an LOS of 2–7 days compared to 1-day increases by 11.1% from ROM level 0 (safe) to level 1, by 33.6% for level 2, by 85.9% for level 3, and by 26.9% for level 4. Similarly, the odds of having an LOS of 8–30 days versus 1-day increases by 19% at ROM level 2, by 107.2% at level 3, and by 81% at level 4. A similar trend was observed with SOI levels, indicating that disease severity measures significantly impact LOS.

For predictive purposes, the model can be used to estimate LOS based on fully known factors such as age, sex, chronic conditions, and disease severity. For example, for a female patient aged ≥ 65 years with MCCs and severe disease severity (ROM = 4, SOI = 4), the probability of having an LOS of 1 day is 4.35%, the probability of 2–7 days is 43.82%, the probability of 8–30 days is 48.01%, and the probability of more than 30 days is 3.82%.

If the same patient had lower severity levels (ROM = 1, SOI = 1), the probability of having an LOS of 1 day would increase to 22.05%, the chance of 2–7 days would rise to 74.04%, and the likelihood of 8–30 days would be just 3.82%, with a very small chance of staying more than a month (0.09%). This highlights the strong impact of disease severity on the expected LOS.

In the absence of complete patient data, we provide predictive results based on various scenarios, averaging the possible outcomes with corresponding prediction errors. For instance, for patients under 65 years of age without MCCs, the probability of having an LOS of 1 day is 10.78%, with 80.90% discharged within 1 week, 17.59% within 8–30 days, and 1.51% for staying more than a month. For patients aged ≥ 65 years with MCCs, these probabilities shift to 12.72%, 76.69%, 21.16%, and 2.15%, respectively. These predicted results are summarized in Table 5.

## 4. Discussion

This study aimed to analyze patients’ hospital length of stay (LOS) and identify the factors influencing it, which is crucial for understanding disease risk and the quality of care provided by medical facilities. The research highlights the relationship between demographic factors (AGE and SEX), multiple chronic conditions (MCCs), and disease severity measures (ROM and SOI) with patients’ LOS.

The findings of this study have important implications for health services. By identifying key factors influencing LOS, hospitals can better allocate resources and plan care. For example, elderly patients with MCCs and severe disease require more intensive inpatient care and post-discharge support. The ability to predict LOS allows healthcare administrators to optimize bed turnover, schedule staffing, and reduce bottlenecks in hospital workflows. Additionally, recognizing that younger patients without MCCs generally have a shorter LOS may help in stratifying patients for targeted interventions.

This study adds value to the existing literature by examining the combined influence of MCCs and disease severity measures on LOS. While prior studies have largely focused on the impact of chronic conditions alone [30,34], this research demonstrates that disease severity (ROM and SOI) is a critical, independent factor influencing LOS. For instance, patients with extreme severity (coded as four) had a mean LOS significantly higher than those with lower severity, even when MCCs were controlled. These results provide a more holistic understanding of LOS determinants, bridging gaps in previous research [21].

Our findings align with previous research indicating that chronic conditions significantly extend LOS [30,34]. However, the inclusion of disease severity measures offers a more comprehensive perspective. Unlike studies that considered these factors in isolation, we identified an interaction between MCCs and disease severity, showing that their combined effect on LOS is greater than either factor alone. Furthermore, the multinomial logistic regression model used in this study provides robust, interpretable results compared to linear regression or Markov chain methods [25], which have been criticized for their complexity or limited applicability.

A key strength of our model lies in its simplicity and user-friendliness, making it adaptable for real-world applications. Healthcare providers can use this model to estimate LOS and plan appropriate care pathways, particularly for high-risk groups. Policymakers can also leverage these insights to design targeted interventions aimed at improving hospital efficiency and patient outcomes. For example, resource-intensive groups, such as elderly patients with MCC, can be prioritized for enhanced care coordination and transitional care programs.

In conclusion, this study demonstrates a significant relationship between demographic factors, MCCs, disease severity measures, and patients’ hospital LOS. Elderly patients with MCCs and high disease severity may have longer lengths of stay, whereas those without MCCs and those with low disease severity tend to have shorter stays, even among older patients. By introducing a practical and robust predictive model, this study provides actionable insights that can enhance hospital operations and improve patient care.

## Figures and Tables

**Table 1:** Patient characteristics in SEX and MCCs (AGE (0 = 18–64 years, 1 = 65 years or older): SEX (0 = male, 1 = female); MCCs, multiple chronic conditions (0 = 0–2 chronic conditions, 1 = 3 chronic conditions or more); ROM, risk of mortality (0 = no class specified, 1 = minor likelihood of dying, 2 = moderate likelihood of dying, 3 = major likelihood of dying, 4 = extreme likelihood of dying); SOI, severity of illness (0 = no class specified, 1 = minor loss of function, 2 = moderate loss of function, 3 = major loss of function, 4 = extreme loss of function).

			SEX = 1 (Female)		MCC = 1	
Variable	Count	Percentage	Count	Percentage	Count	Percentage
**Total**	748,572	100.00	435,642	58.20	499,534	66.73
**AGE**						
0: under 65	427,045	57.05	260,954	61.11	208,107	48.73
1: no less than 65	321,527	42.95	174,688	54.33	291,427	90.64
**ROM**						
0	195	0.03	89	45.64	62	31.79
1	380,785	50.87	248,053	65.14	171,719	45.10
2	181,864	24.29	93,925	51.65	157,444	86.57
3	138,419	18.49	70,743	51.11	127,725	92.27
4	47,309	6.32	22,832	48.26	42,584	90.01
**SOI**						
0	195	0.03	89	45.64	62	31.79
1	207,703	27.75	137,726	66.31	85,165	41.00
2	304,162	40.63	175,835	57.81	208,965	68.70
3	187,303	25.02	98,573	52.63	161,800	86.38
4	49,209	6.57	23,419	47.59	43,542	88.48

**Table 2:** LOS distribution among patient characteristics.

Variable	Count				Percentage			
	LOS = 1	LOS = 2	LOS = 3	LOS = 4	LOS = 1	LOS = 2	LOS = 3	LOS = 4
**Total**	98,848	536,971	106,937	5816	13.20	71.73	14.29	0.78
**AGE**								
0: under 65	61,790	311,424	50,221	3610	14.47	72.93	11.76	0.85
1: no less than 65	37,058	225,547	56,716	2206	11.53	70.15	17.64	0.69
**SEX**								
0: Male	45,919	210,183	53,654	3174	14.67	67.17	17.15	1.01
1: Female	52,929	326,788	53,283	2642	12.15	75.01	12.23	0.61
**MCC**								
0	36,167	189,976	21,212	1683	14.52	76.28	8.52	0.68
1	62,681	346,995	85,725	4133	12.55	69.46	17.16	0.83
**ROM**								
0	32	131	29	3	16.41	67.18	14.87	1.54
1	66,061	285,150	27,735	1839	17.35	74.88	7.28	0.48
2	22,249	134,823	23,997	795	12.23	74.13	13.20	0.44
3	8044	95,130	34,131	1114	5.81	68.73	24.66	0.80
4	2462	21,737	21,045	2065	5.20	45.95	44.48	4.36
**SOI**								
0	32	131	29	3	16.41	67.18	14.87	1.54
1	43,671	154,845	8796	391	21.03	74.55	4.23	0.19
2	41,830	230,366	30,406	1560	13.75	75.74	10.00	0.51
3	11,164	131,293	43,615	1231	5.96	70.10	23.29	0.66
4	2151	20,336	24,091	2631	4.37	41.33	48.96	5.35

**Table 3:** Results of the multinomial logistic regression model.

Coefficient	Estimate			95% Confidence Interval Timate			*p*-Value		
(*β*)	i=2	i=3	i=4	i=2	i=3	i=4	i=2	i=3	i=4
$\beta_{i}^{\left(0\right)}$	1.343	−0.083	−2.108	0.955;	−0.586;	−3.293;	<0.001	0.037	<0.001
				1.730	0.420	−0.923			
$\beta_{i}^{\left(age\right)}$	−0.022	−0.156	−0.682	−0.039;	−0.178;	−0.745;	0.001	<0.001	<0.001
				−0.005	−0.134	−0.619			
$\beta_{i}^{\left(sex\right)}$	0.372	0.110	−0.082	0.358;	0.091;	−0.137;	<0.001	<0.001	<0.001
				0.386	0.128	−0.027			
$\beta_{i}^{\left(mcc\right)}$	−0.275	−0.099	−0.427	−0.292;	−0.123;	−0.494;	<0.001	<0.001	<0.001
				−0.259	−0.075	−0.359			
$\beta_{i1}^{\left(rom\right)}$	0.105	−0.061	0.189	−0.090;	−0.314;	−0.407;	0.015	0.032	0.027
				0.300	0.191	0.785			
$\beta_{i2}^{\left(rom\right)}$	0.290	0.174	−0.182	0.095;	−0.079;	−0.779;	<0.001	0.009	0.028
				0.485	0.426	0.415			
$\beta_{i3}^{\left(rom\right)}$	0.620	0.728	0.090	0.425;	0.476;	−0.507;	<0.001	<0.001	0.038
				0.816	0.981	0.687			
$\beta_{i4}^{\left(rom\right)}$	0.238	0.593	0.253	0.038;	0.337;	−0.346;	0.001	<0.001	0.020
				0.438	0.849	0.853			
$\beta_{i1}^{\left(soi\right)}$	−0.311	−1.463	−2.437	−0.506;	−1.716;	−3.039;	<0.001	<0.001	<0.001
				−0.116	−1.210	−1.835			
$\beta_{i2}^{\left(soi\right)}$	0.155	−0.238	−0.730	−0.039;	−0.490;	−1.326;	0.006	0.003	0.001
				0.350	0.015	−0.134			
$\beta_{i3}^{\left(soi\right)}$	0.753	1.099	0.599	0.558;	0.846;	0.004;	<0.001	<0.001	0.002
				0.948	1.351	1.195			
$\beta_{i4}^{\left(soi\right)}$	0.655	2.037	2.918	0.455;	1.78;	2.319;	<0.001	<0.001	<0.001
				0.856	2.293	3.516			

**Table 4:** Estimates of odds ratios using the multinomial logistic regression model (LOS = 1 was considered as the reference category): AGE (0 = 18–64 years, 1 = 65 years or older); SEX (0 = male, 1 = female); MCC, multiple chronic conditions (0 = 0–2 chronic conditions, 1 = 3 chronic conditions or more); ROM, risk of mortality (0 = no class specified, 1 = minor likelihood of dying, 2 = moderate likelihood of dying, 3 = major likelihood of dying, 4 = extreme likelihood of dying); SOI, severity of illness (0 = no class specified, 1 = minor loss of function, 2 = moderate loss of function, 3 = major loss of function, 4 = extreme loss of function); LOS, hospital length of stay (1 = 1 day, 2 = 2–7 days, 3 = 8–30 days, 4 = 31 days or more).

Variable	Estimate of Odds Ratio		
	LOS = 2	LOS = 3	LOS = 4
	OR (95% CI)	OR (95% CI)	OR (95% CI)
AGE = 1	0.978 (0.962, 0.995)	0.856 (0.837, 0.875)	0.506 (0.475, 0.539)
SEX = 1	1.451 (1.431, 1.471)	1.116 (1.095, 1.137)	0.921 (0.872, 0.973)
MCC = 1	0.759 (0.747, 0.772)	0.905 (0.884, 0.927)	0.652 (0.610, 0.698)
ROM = 1	1.111 (0.914, 1.350)	0.941 (0.731, 1.211)	1.208 (0.665, 2.192)
ROM = 2	1.336 (1.099, 1.624)	1.190 (0.924, 1.532)	0.834 (0.459, 1.514)
ROM = 3	1.859 (1.529, 2.261)	2.072 (1.609, 2.668)	1.094 (0.602, 1.987)
ROM = 4	1.269 (1.039, 1.549)	1.810 (1.401, 2.338)	1.288 (0.707, 2.347)
SOI = 1	0.733 (0.603, 0.891)	0.231 (0.180, 0.298)	0.087 (0.048, 0.160)
SOI = 2	1.168 (0.961, 1.420)	0.788 (0.613, 1.015)	0.482 (0.265, 0.875)
SOI = 3	2.123 (1.747, 2.581)	3.000 (2.331, 3.861)	1.821 (1.004, 3.303)
SOI = 4	1.926 (1.576, 2.353)	7.665 (5.931, 9.907)	18.499 (10.168, 33.659)

**Table 5:** Predicted probabilities for LOS based on patient characteristics among MCCs (calculation unit: %): AGE (0 = 18–64 years, 1 = 65 years or older); SEX (0 = male, 1 = female); MCC, multiple chronic conditions (0 = 0–2 chronic conditions, 1 = 3 chronic conditions or more); ROM, risk of mortality (0 = no class specified, 1 = minor likelihood of dying, 2 = moderate likelihood of dying, 3 = major likelihood of dying, 4 = extreme likelihood of dying); SOI, severity of illness (0 = no class specified, 1 = minor loss of function, 2 = moderate loss of function, 3 = major loss of function, 4 = extreme loss of function); LOS, hospital length of stay (1 = 1 day, 2 = 2–7 days, 3 = 8–30 days, 4 = 31 days or more).

Patient Characteristics		Predicted Probabilities (Prediction Error) for Patients’ LOS			
MCC	Variable	LOS = 1	LOS = 2	LOS = 3	LOS = 4
MCC = 0	AGE = 0	10.78 (1.71)	70.12 (3.24)	17.59 (3.70)	1.51 (0.65)
	AGE = 1	10.30 (1.70)	68.04 (3.66)	18.97 (3.75)	2.69 (1.12)
	SEX = 0	12.02 (1.87)	65.92 (3.43)	19.63 (3.87)	2.42 (1.05)
	SEX = 1	9.06 (1.39)	72.24 (3.24)	16.93 (3.53)	1.77 (0.79)
	ROM = 0	12.92 (1.93)	67.46 (3.23)	17.20 (3.53)	2.42 (1.06)
	ROM = 1	12.05 (1.78)	69.95 (3.17)	15.24 (3.19)	2.77 (1.22)
	ROM = 2	10.29 (1.54)	71.63 (3.14)	16.44 (3.48)	1.64 (0.73)
	ROM = 3	7.44 (1.19)	71.24 (3.70)	19.86 (3.96)	1.46 (0.64)
	ROM = 4	10.01 (1.62)	65.14 (3.89)	22.66 (4.24)	2.19 (0.92)
	SOI = 0	12.51 (0.89)	71.43 (1.28)	14.87 (0.92)	1.19 (0.16)
	SOI = 1	18.26 (1.24)	76.53 (1.27)	5.06 (0.35)	0.15 (0.02)
	SOI = 2	11.57 (0.84)	77.04 (1.10)	10.85 (0.71)	0.53 (0.07)
	SOI = 3	5.96 (0.46)	71.92 (1.31)	21.09 (1.20)	1.03 (0.14)
	SOI = 4	4.40 (0.33)	48.49 (1.77)	39.52 (1.57)	7.58 (0.92)
MCC = 1	AGE = 0	13.31 (2.09)	65.90 (3.22)	19.59 (4.05)	1.20 (0.52)
	AGE = 1	12.72 (2.07)	63.97 (3.57)	21.16 (4.13)	2.15 (0.90)
	SEX = 0	14.75 (2.27)	61.54 (3.29)	21.77 (4.25)	1.93 (0.84)
	SEX = 1	11.28 (1.73)	68.33 (3.21)	18.97 (3.89)	1.42 (0.63)
	ROM = 0	15.86 (2.34)	63.07 (3.12)	19.14 (3.91)	1.93 (0.84)
	ROM = 1	14.88 (2.17)	65.81 (3.06)	17.08 (3.56)	2.23 (0.98)
	ROM = 2	12.75 (1.90)	67.54 (3.11)	18.39 (3.84)	1.32 (0.58)
	ROM = 3	9.28 (1.48)	67.38 (3.71)	22.18 (4.32)	1.17 (0.50)
	ROM = 4	12.31 (1.99)	60.88 (3.78)	25.07 (4.61)	1.74 (0.73)
	SOI = 0	15.43 (1.06)	67.01 (1.37)	16.61 (1.00)	0.95 (0.13)
	SOI = 1	22.47 (1.45)	71.76 (1.45)	5.65 (0.39)	0.12 (0.02)
	SOI = 2	14.40 (1.01)	72.94 (1.23)	12.24 (0.78)	0.43 (0.06)
	SOI = 3	7.41 (0.56)	68.01 (1.39)	23.74 (1.30)	0.83 (0.11)
	SOI = 4	5.38 (0.41)	44.96 (1.71)	43.62 (1.63)	6.04 (0.74)

## Data Availability

Data are contained within the article.

## Appendix A

The variables in this study are defined as follows:

- AGE: This variable represents the patient’s age in years, and only individuals aged 18 or older were included. To analyze differences in hospital length of stay (LOS) among elderly patients, AGE was categorized into two groups: under 65 years (coded as 0) and 65 years or older (coded as 1).
- SEX: this variable indicates the patient’s sex, with males coded as 0 and females as 1.
- ROM (risk of mortality): This variable is divided into five levels (0–4). The levels are categorized as follows: (0) no classification, (1) minor risk of death, (2) moderate risk, (3) major risk, and (4) extreme risk.
- SOI (severity of illness): this variable is also divided into five levels (0–4) and categorized as follows: (0) no classification, (1) minor functional loss, (2) moderate loss, (3) major loss, and (4) extreme loss.
- MCC: This variable identifies patients with multiple chronic conditions. The classification method for chronic diseases follows previous research [1,35], categorizing diseases into 46 major groups using the International Classification of Diseases, 10th Revision, Clinical Modification [36]. For example, conditions with ICD-10-CM codes F00–03, F05.1, G30, G31, and R54 are grouped under “dementia”. Patients are classified as having MCCs if they have at least three chronic conditions. MCCs are coded as 0 if fewer than three conditions are present and 1 otherwise.
- LOS: This variable represents hospital length of stay. Instead of treating LOS as a continuous variable, it is categorized for analysis into four groups: 1 day, 2–7 days, 8–30 days, and more than 30 days. This classification aligns with previous studies [21,22,30].
